# Anatomical and clinical significance of the Cyrano long-nosed patella in combination with patellofemoral instability: a case report and review of literature

**DOI:** 10.1186/s13256-025-05235-w

**Published:** 2025-04-23

**Authors:** Kunpeng Yang, Andreas Prescher, Frank Hildebrand, Christian David Weber

**Affiliations:** 1 Department of Orthopaedic, Trauma, and Reconstructive Surgery, RWTH University Hospital, Pauwelsstraße 30, 52074 Aachen, Germany; 2 Department of Orthopaedics, The Third People’s Hospital of Henan Province, Funiu Road 198, Zhengzhou, 450000 China; 3 Department of Anatomy, RWTH University Hospital, Pauwelsstraße 30, 52074 Aachen, Germany

**Keywords:** Long-nosed/Cyrano patella, Patella alta, Patella instability, Osgood–Schlatter disease, TTO, Dynamic MPFL reconstruction

## Abstract

**Background:**

The combination of a long-nosed patella and patella alta can lead to symptoms such as anterior knee pain and patellofemoral instability.

**Purpose:**

Our objective was to address this uncommon, multifactorial cause of patellar pain and instability by a single-stage combined surgical approach.

**Case presentation:**

A 14-year-old German female presented to our hospital for recurrent patellar dislocations and exacerbated infrapatellar pain during kneeling. Following physical examination and imaging, the patient was diagnosed with patellar instability combined with the rare “Cyrano”-type patella. The patient underwent a single-stage procedure that included knee arthroscopic exploration, inferior pole osteotomy of the patella, tibial tuberosity osteotomy, and dynamic medial patellofemoral ligament reconstruction. Following the surgical procedure, the symptoms of knee pain and instability were entirely alleviated, accompanied by notable enhancements in the Knee Injury and Osteoarthritis Outcome Score , Kujala Score, and Lysholm Score. Notably, no recurrence was observed throughout the 2-year follow-up period.

**Conclusion:**

The entity of a symptomatic Cyrano patella may be combined with patellofemoral instability. Osteotomy and surgical excision of the long-nosed aspect of the inferior pole, coupled with the realignment of the patellofemoral joint in both coronal and sagittal planes, alleviated pain and enhanced knee joint stability, ultimately contributing to the resolution of this uncommon condition. A sagittal plane deformity of the patellar shape may significantly affect the measurement of the patella height, as the Insall–Salvati index may not correctly determine the height of a patella owing to the elongated nose.

*Level of evidence* IV.

## Introduction

**Fig. 1:**
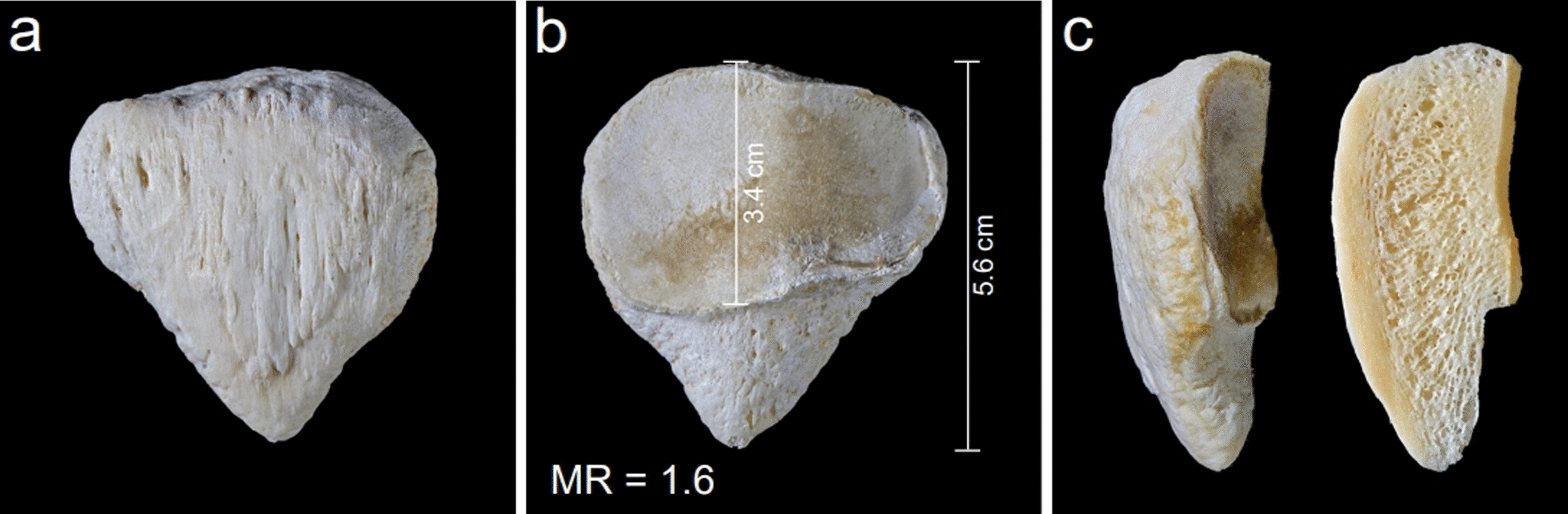
Typical left Cyrano patella. **a** Ventral aspect of a typical Cyrano patella. **b** Dorsal aspect of the same specimen. **c** Lateral aspect and midsagittal section of the specimen

Grelsamer et al. [9] introduced a classification of the patella according to the sagittal shape. The authors differentiate three types and state that types II and III are often combined with patellar pain. Type II patella is characterized by a long inferior pole and a shortened articular facet. For this type, Grelsamer introduced the term “Cyrano’s long-nosed patella.” This term refers to the verse drama “Cyrano de Bergerac” written by Edmond Rostand in 1897. The knight Cyrano was characterized by an abnormally long and pointed nose. The Cyrano patella was also characterized by Grelsamer with the morphology ratio (MR). This ratio describes the relationship of the patellar length (Fig. 1a) to the length of the articular surface (Fig. 1b). This ratio a/b is greater than 1.5 in the Cyrano patella. A distinctive feature of Cyrano patella is its elongated inferior pole (> 14 mm but < 27 mm), making up 6% of cases in the patellar nose classification among North Indians [1]. The Cyrano patella is not a new description by Grelsamer, it is nicely figured with an X-ray in Alban Köhler’s book “Grenzen des Normalen und Anfänge des Pathologischen im Röntgenbilde” and described as cone-shaped (zapfenförmig) or beak-shaped (schnabelförmig) patella anomaly [17]. In the 13 th edition, this anomaly is not further mentioned.

Recent reports show an increasing trend in incidences of patellar dislocation over the past decade and the majority of affected individuals are adolescents between 10 and 19 years old [8]. Furthermore, a significant proportion of dislocations are attributed to causative factors associated with athletic sports activities. Various factors, including patella alta, knee valgus, and femoral trochlea dysplasia, can cause patellar instability, which may arise from one or multiple factors in clinical practice. Current literature does not characterize the Cyrano patella and its relevance in patellofemoral instability. Consequently, we successfully managed a rare case of Cyrano patella with multiple risk factors for instability. In addition, we investigate the progression of this condition through a single-staged combined surgery and mid-term follow-up.

### Anatomical analysis of patella morphology

**Fig. 2:**
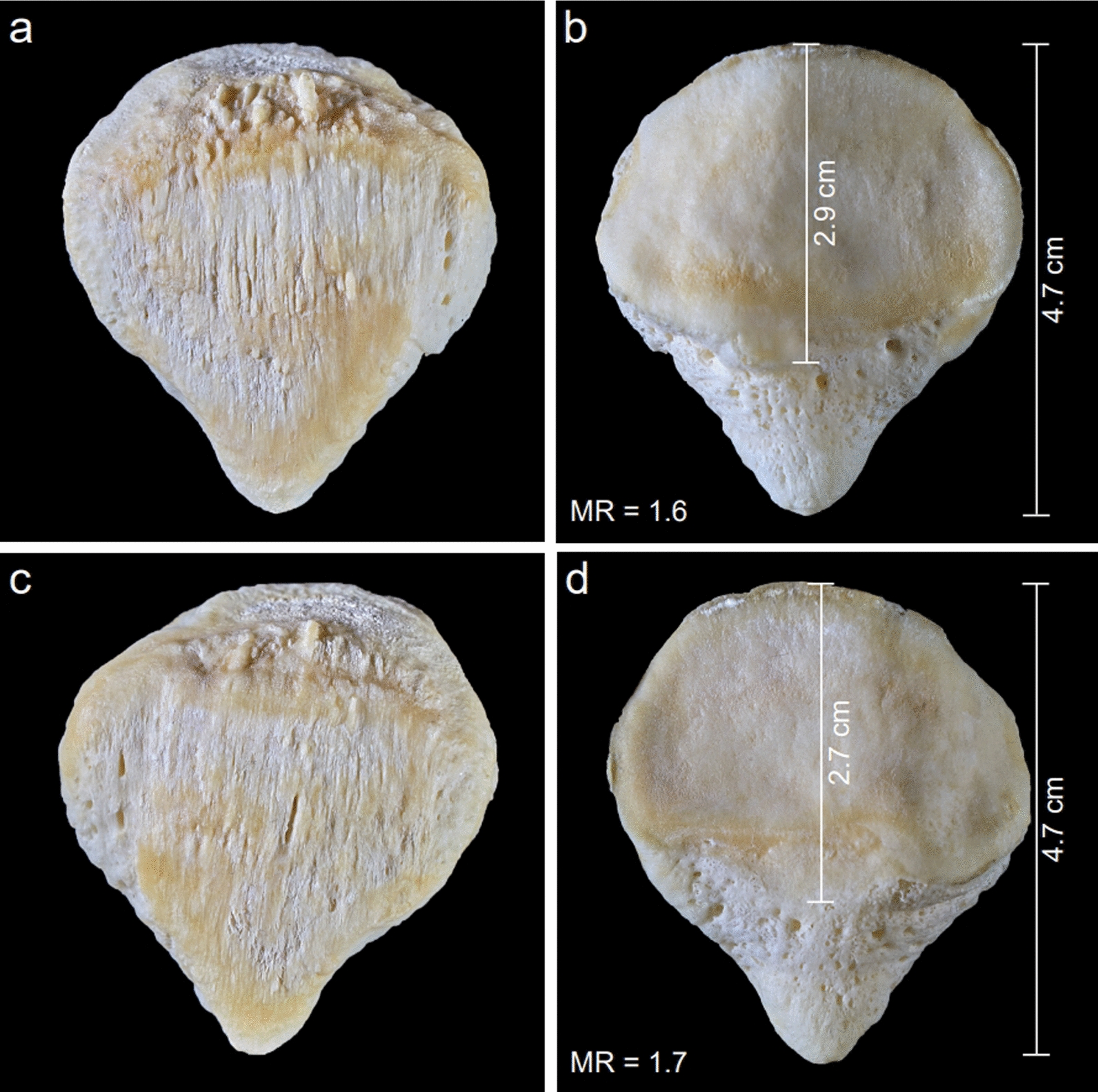
Absolute symmetrical occurrence of a typical bilateral Cyrano patella in a 67-year-old male. **a** Ventral aspect of the right Cyrano patella. **b** Dorsal aspect of the right Cyrano patella. **c** Ventral aspect of the left Cyrano patella. **d** Dorsal aspect of the left Cyrano patella

**Fig. 3:**
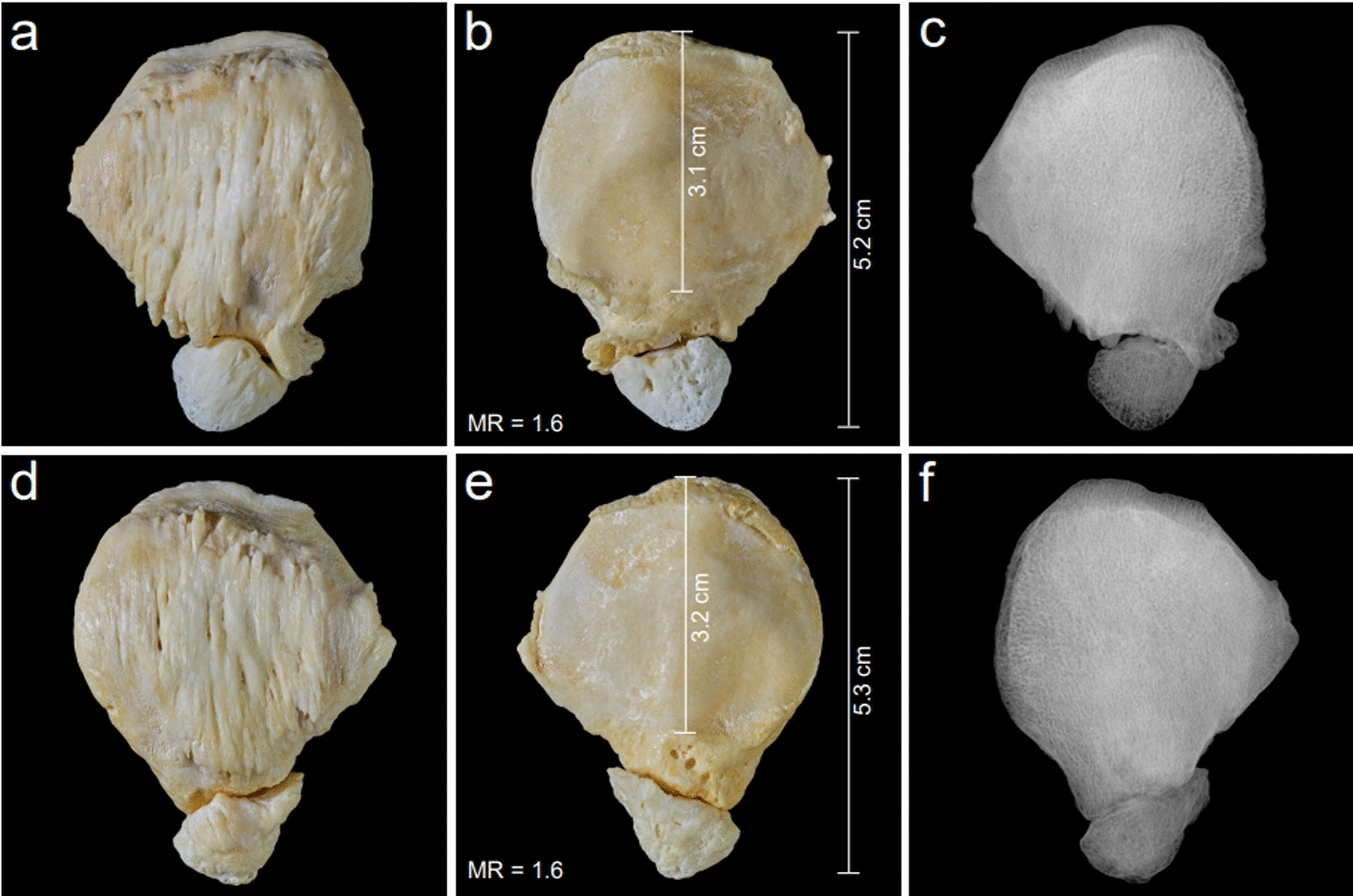
Symmetric occurrence of a patella bipartita Saupe type I in a 63-year-old female. **a** Ventral aspect of the right specimen. **b** Dorsal aspect of the right specimen. **c** X-ray of the right specimen. **d** Ventral aspect of the left specimen. **e** Dorsal aspect of the left specimen. **f** X-ray of the left specimen

Figure 1 shows a typical anatomical specimen of such a Cyrano patella with a morphology ratio of 1.6. The condition usually shows no pathological osseous processes, so it cannot be interpreted as an osteophyte or another pathological appearance. It must be assumed that it is an inborn anatomical variation. Figure 2 presents another bilaterally expressed, absolutely symmetrical case belonging to a 67-year-old male with MR = 1.6 on the right side and MR = 1.7 on the left side. In the context of the Cyrano patella, another anatomical variation of the inferior patellar pole must be considered: the rare patella bipartita Saupe type I. This variation is characterized by an isolated ossicle forming the inferior pole. These patellae also often present an enlarged, lengthened inferior pole and a greater craniocaudal length with a reduced length of the articular facet and an MR also greater than 1.5. Therefore a striking similarity to the Cyrano patella is obvious. Figure 3 presents such a bilaterally expressed specimen belonging to a 63-year-old female together with the X-rays of these specimens. The MR is determined as 1.6 for the right side as well as for the left side. It can be assumed that these two abnormalities of the inferior pole (Cyrano patella and patella bipartita Saupe type I) are parts of a teratological series and, therefore, are inborn occurrences. But it must also be considered that these two variations may be caused by increased stress to the inferior pole during the growth phase. This explanation was also considered by Joachimsthal [14] for his case of atypical patella bipartita Saupe type I found in a child suffering from “Little’s disease” (spastic cerebral palsy) with flexion contracture. Considering this explanation, due to increased traction stress, it would be possible that, in normal ossifications of the patella, a Cyrano patella can develop owing to the increased stress on the growing inferior pole. But if there is a secondary ossification center at the inferior pole, for example, as described by Schaer *et al*. [25], it can be separated by such an abnormal traction force and cause a patella bipartita Saupe type I. Differential diagnostics must rule out Sinding-Larsen-Johannson disease, characterized by irregular small ossicles and defects located at the inferior patellar pole, which is also not enlarged. The rare patella bipartita Saupe type I was observed by Riess [23], also in a case of Little’s disease, and interpreted as a stress fracture. This explanation does not seem convincing because only a minor amount of fibers are inserted at the inferior pole. Hempfling *et al*. [10] stressed also the major role of disturbed ossification in the development of a patella partita and named it “Apophysen-Äquivalent”. All the different hypotheses concerning the development of the patella partita are discussed and enumerated by Olson and cannot be discussed in this paper encyclopedically. Because there is an obvious similarity between the Cyrano patella and patella partita Saupe type I, it is suggested to use the classification Cyrano patella type I for the cone-shaped appearance and the designation Cyrano patella type II for the one characterized by the isolated inferior pole ossicle.

## Case presentation

Patient information: The patient was a 14-year-old German female student weighing 110 kg and standing 179 cm tall with a body mass index of 34.3 kg/m^2^. She actively participates in extracurricular dance.

Medical history: Treatment included conservative therapy for suspected Osgood–Schlatter disease and management of irritable bowel syndrome.

Chief complaint: The patient experienced significant infrapatellar pain while kneeling and recurrent external patellar dislocations, though she reported no discomfort during knee flexion or extension.

Preoperative knee-related scores: The Kujala Score was 32, and the Lysholm Score was 19. Knee Injury and Osteoarthritis Outcome Score (KOOS) Pain subscale was 21.4, KOOS Symptoms subscale was 58.3, KOOS Activities of Daily Living subscale was 41.2, KOOS Sports/Recreation subscale was 10, and KOOS Quality of Life subscale was 25.

### Clinical examination

Physical examination: The left knee did not exhibit significant pressure pain. However, tests for patellar trajectory and apprehension were positive. Tests for internal and external rotation stress, anterior and posterior drawer, and patellofemoral grinding were negative. The range of motion (ROM) was within normal limits.

Laboratory examinations: All biochemical analyses, encompassing albumin levels, electrolyte balances, and transaminase activities, along with blood assessments that comprised evaluations of red blood cell counts, white blood cell counts, platelet counts, and hemoglobin concentrations, as well as inflammatory marker assessments, including measurements of C-reactive protein and interleukin levels, yielded results that fell within the established normal ranges.

Diagnostic imaging: Preoperative lateral patellar height assessment for the left knee at 20° flexion used four common patellar height ratios (Table 1). Preoperative computed tomography (CT) scan: measurements for the left knee included a tibial tubercle–trochlear groove (TT–TG) distance of 21.1 mm, patellar tilt of 36.5°, femoral torsion of 13.6°, and tibial torsion of 30.2° (Table 2). Preoperative magnetic resonance imaging (MRI): the scan measured the left knee’s sulcus angle at 129.3° and trochlear depth at 6.4 mm (Table 2) and found no significant meniscus injuries or ligament ruptures.

**Table 1:** Patella height assessment with different methods of the left knee

Measurement methods	Calculated parameters	Values
Caton–Deschamps ratio	b/a	1.7
Insall–Salvati ratio	d/c	0.8
Modified Insall–Salvati ratio	f/e	2.3
Blackburne–Peel ratio	h/g	1.6

**Table 2:** Preoperative measurement parameters of the left knee

Measurement parameters	Preoperative values
TT–TG Distance	21 mm
Patella tilt	36.5°
Femoral torsion	13.6°
Tibial torsion	30.2°
Sulcus angle	129.3°
Morphology ratio	2

Diagnostic assessment: On the basis of the findings, we diagnosed type 3b-c patella instability and maltracking according to Frosch and Schmeling [8].

### Management and outcome

**Fig. 4:**
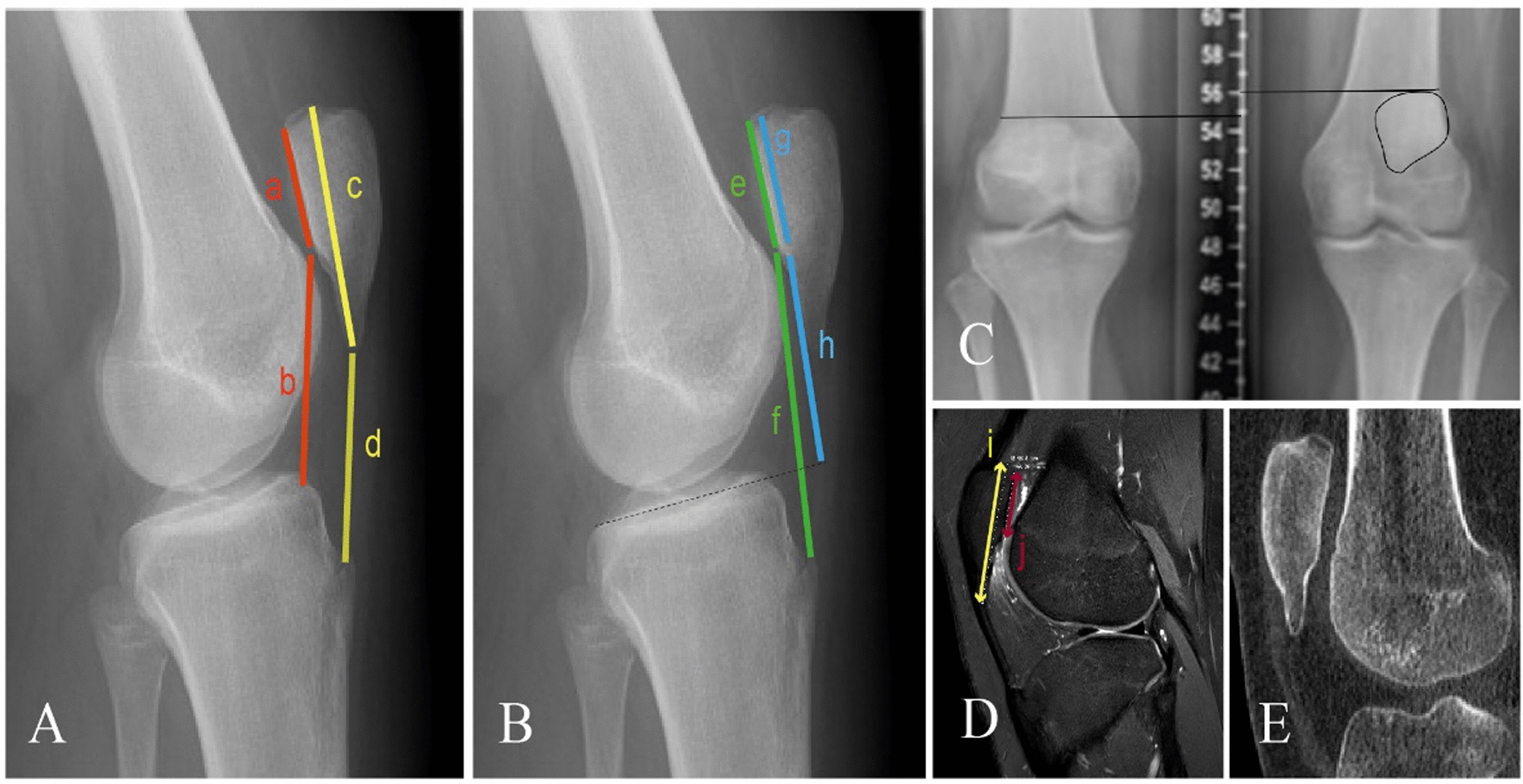
**A** The solid black lines indicate the surface projections of the actual patellar and tibial tuberosity positions, and the dashed lines indicate the projected postoperative surface projections of the patella, patellar tendon, and tibial tuberosity. **B** The long nose (inferior pole of the patella) was resected and it was approximately 17 mm in length without articular cartilage attachment. **C** Intraoperative view shows that the tibial tubercle osteotomy involves distalization and medialization; two screws are well positioned in the fragment. **D** The repeat arthroscopic exploratory procedure shows the patellar trajectory aligns centrally within the femoral trochlear during both flexion and extension movements; *yellow arrow* sagittal dimension, *red arrow* articular surface. **E** Dynamic medial patellofemoral ligament reconstruction technique involves the gracilis tendon, which was threaded around the incised sartorius fascia and then passed beneath the skin to reach the medial patellar margin

Surgical procedure: The patient, positioned supine with an arthroscopic leg holder, underwent the procedure under a pneumatic tourniquet. General anesthesia was administered along with a peripheral femoral nerve block. During the examination, which was under anesthesia, a tendency for dislocation was observed up to a 45° flexion, with no additional signs of instability. Initial diagnostic arthroscopy showed no remarkable cartilage damage. However, it did reveal a patella laterally displaced and prone to dislocation upon applying pressure with two fingers. Further exploration revealed a dysplastic trochlea and a laterally displaced patella in the upper recess. No cartilage damage was observed on the patella or femoral gliding surface. In addition, no notable abnormalities were detected in the lateral or medial recesses. The menisci and cruciate ligaments were free of pathological findings. A cutaneous incision provided longitudinal access to expose the patella. Performing an arthrotomy via a paramedian lateral approach with a lateral release exposed the inferior patellar slope. The tip of the patella was in contact posterior to the patellar ligament. The distal process was resected using oscillation while protecting and preserving the patellar ligament. The removed fragment measured 17 mm in length (Fig. 4B).

**Fig. 5:**
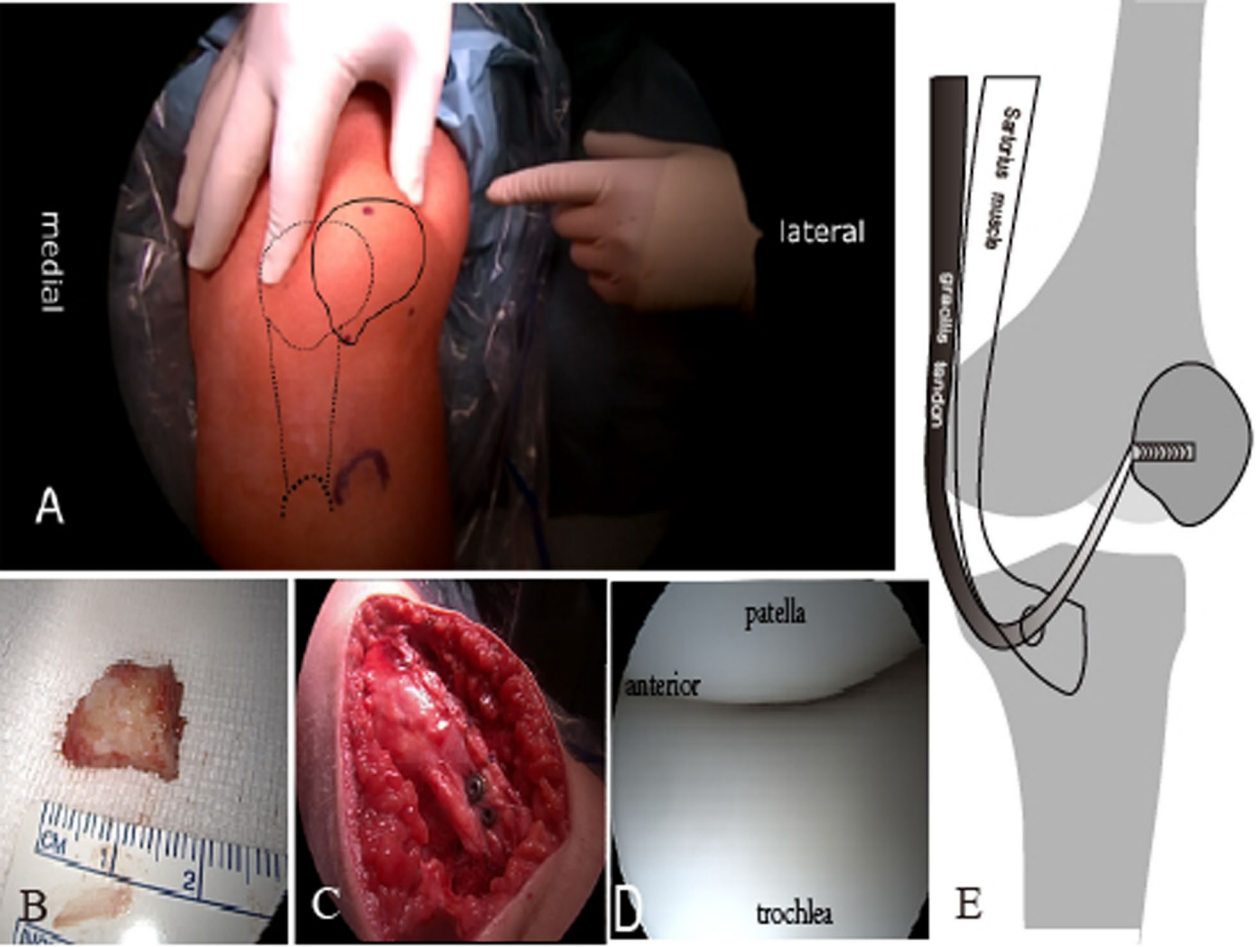
**A** A postoperative anterior view radiograph taken in the operating room indicated that two screws had cross-fixed the tibial tubercle. **B** A postoperative lateral radiograph of the left knee, also taken in the operating room, showed a substantial reduction in patellar height; the Caton–Deschamps index had decreased from 1.7 preoperatively to 1 postoperatively. **C** A lateral radiograph taken at the 2-year follow-up postoperatively still maintained a Caton–Deschamps ratio (a/b) of 1 and an morphology ratio (c/a) of 1.4. **D** The 2-year postoperative follow-up revealed that the flexion angle was 120° and the extension angle was 0°** E**

Next, the gracilis tendon was sought for dynamic medial patellofemoral ligament reconstruction (Fig. 4E). The tendon was dissected carefully, looped distally, and reinforced with a fiber wire suture. Following this, we proceeded with the tibial tuberosity osteotomy (TTO) in standard fashion. The tuberosity was repositioned medially by approximately 10 mm and distally by 15 mm (Fig. 4A). Fixation was achieved with two cannulated screws and a proximal washer (Königsee Implantate GmbH, Allendorf, Germany). This step satisfactorily corrected the patellar trajectory within the trochlea (Fig. 4C). Subsequently, medial patellar anchoring was performed by palpating the patella’s groove along the target wire at the patellar edge. A hollow drill was used to over-drill the wire, followed by the insertion of the reinforced suture end into the blind hole with a 4.75 mm Swivelock anchor (Arthrex, Inc., Naples, Florida, USA) to secure it. To further minimize patellar tilt, fiber wire sutures from the Swivelock were used for medial retraction. This enabled full knee joint extension and flexion. As a result, the patella no longer laterally shifted or tended toward dislocation. Finally, arthroscopy was performed again to verify patellar alignment (Fig. 4D). Fascial stitching, followed by subcutaneous and skin sutures, concluded with the application of a sterile bandage and wrapping of the leg. Fluoroscopy confirmed the tibial tubercle was well-positioned by two screws (Fig. 5A), with the patellar height close to the normal range in the operating room (Fig. 5B).

### Postoperative rehabilitation program

The recovery plan focused on intensive vastus medialis oblique strengthening and bracing, supported by 6 weeks of hard frame orthosis use. For the first 6 weeks, partial weight-bearing of 20 kg was recommended, with a progressive movement protocol increasing knee flexion from 30° to 90° over the period.

### Outcome and follow-up

The patient showed no local complications, including incision infection or fat liquefaction nor any lower extremity venous thrombosis post surgery. A 2-year postoperative X-ray review of the left knee showed the Caton–Deschamps ratio (a/b = 1) and the MR (c/a = 1.4) remained within the normal range (Fig. 5C). On the latest assessment, the patient exhibited no knee pain while kneeling and no further episodes of patellar dislocation; she was able to return to dancing activities. The ROM in the left knee was measured at 0–120° (Fig. 5D-E). Knee-related outcome evaluations revealed a Kujala Score of 94, Lysholm Score of 86, KOOS Pain subscale at 94.4, KOOS Symptoms subscale at 92.9, KOOS Activities of Daily Living subscale at 100, KOOS Sports/Recreation subscale at 95, and KOOS Quality of Life subscale at 93.6.

## Discussion

A recent archaeological record indicated the presence of the “Cyrano” patella dating back to the Migration Period in the fifth century AD [20]. The atypical patella morphology could potentially result in various symptoms, including anterior knee pain and functional impairment [16]. Infrapatellar pain and patellar instability are caused by multiple factors. We consider that kneeling pain primarily results from the prolonged inferior pole of the patella irritating the knee’s anterior soft tissues. Intraoperative findings confirmed our suspicion that the patellar tip directly affects the patellar ligament’s integrity (Fig. 4B–C). The mechanism for anterior knee pain in this case closely mirrors patellar tendinopathy, or Jumper’s knee. This condition results from pathological inflammation at the enthesis due to overuse [5]. While both conditions cause anterior knee pain, the underlying factors differ. Jumper’s knee is triggered by activities such as exercise or prolonged knee bending, such as vertical jumping. However, long-nosed subpatellar pain is induced by kneeling, and typical flexion and extension of the knee do not elicit pain. Furthermore, while abnormal bony prominence of the inferior pole of the patella exists in cases of jumper’s knee, it is not as prominent as observed in the long-nosed patella. As seen in our patient, the distal resection of 17 mm exceeded the amount typically observed in cases of Jumper’s knee.

With respect to the etiology of the Cyrano-type patella in the reported patient, both hypotheses of a congenital and acquired variant due to mechanical stress are worth discussing. We identified a positive family history for patellofemoral pain in both the mother and the sister of the patient; however, the patellae morphology ratios of both relatives were normal. Furthermore, the increased body mass index (BMI) could support the hypothesis that mechanical stress may have played a role in the development of this specific patella configuration. Future epidemiologic and biomechanical studies are necessary in this context.

Recurrent patellar dislocation and pain serve as manifestations of patellofemoral instability. Meanwhile, several risk factors contribute to patellofemoral instability, encompassing bony elements such as pathological TT–TG, tibial or femoral torsion deformities, femoral trochlear dysplasia, patella alta, as well as soft tissue factors such as ligament tears and capsular contractures. A novel classification system published by Frosch *et al*. [7, 8] categorized patellofemoral instability and maltracking into five types and has been established on the basis of a comprehensive analysis of patient history, clinical examination, and radiological assessment. This classification aids in implementing a standardized treatment protocol. Type 1 involves simple traumatic dislocations without maltracking or instability. Type 2 features instability without maltracking, often treated with surgical stabilization such as medial patellofemoral ligament (MPFL) augmentation. Type 3 presents with both instability and maltracking, potentially due to soft tissue contractures, muscular deficiencies, patella alta, abnormal TT–TG distance, genu valgum, or torsional deformities, requiring comprehensive treatment addressing both soft tissue and bony anomalies. Type 4 involves severe trochlear dysplasia with loss of patella tracking, typically necessitating trochleaplasty. Type 5 pertains to maltracking without instability, often observed in patients with underlying pathological conditions or specific torsional deformities. Notably, the typing does not include the patellar deformity, so this case offers a valuable reference for the potential inclusion of subtypes in future considerations.

**Fig. 6:**
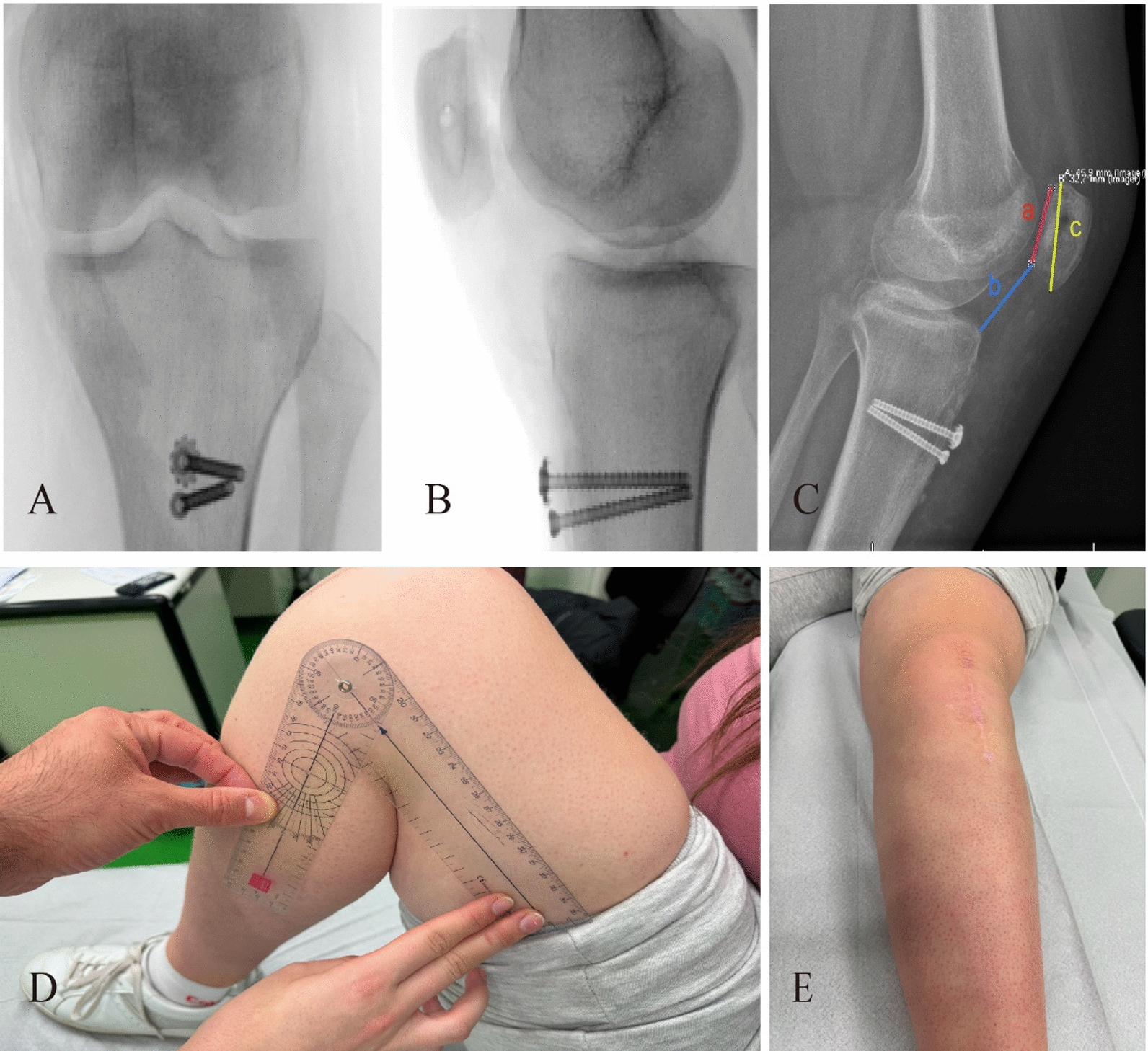
** A** The preoperative lateral radiograph of the left knee with a patellar Caton–Deschamps index (b/a) of 1.7 and Insall–Salvati index (d/c) of 0.8. **B** The preoperative lateral radiograph of the left knee with a patellar modified Insall–Salvati index (f/e) of 2.3 and Blackburne–Peel index of 1.6. **C** Preoperative anteroposterior view radiograph of the bilateral knee showed that the height of the patella in the left knee was 15 mm higher than that in the right knee (indicated by the black lines). **D** Preoperative sagittal plane magnetic resonance imaging revealed a morphology ratio (i/j) of 2. **E** The preoperative sagittal plane of the computed tomography scan

The establishment of the surgical protocol was guided by pertinent preoperative imaging measurements. Various measurement methods are employed to assess patellar height, with imaging modalities remaining the predominant protocol. These methods encompass the Insall–Salvati ratio (IS), Blackburne–Peel ratio (BP), Caton–Deschamps ratio (CD), modified Insall–Salvati ratio (MIS), and patellotrochlear index (PTI). Regrettably, there is currently no consensus in literature regarding the preferred measurement method or an agreed upon cutoff value [30]. To maximize the possibility of avoiding overestimation or underestimation of patellar height, the patient was evaluated and analyzed preoperatively using four commonly used patellar height ratios, with measurements of 1.72 for the Caton–Deschamps index, 0.8 for the Insall–Salvati ratio index (Fig. 6A), 2.3 for the modified Insall–Salvati ratio index, and 1.6 for the Blackburne–Peel index (Fig. 6B), respectively. Verhulst *et al*. [30] propose that utilizing the Insall–Salvati ratio obtained from radiographs is a reliable approach to evaluate patella height. However, our preoperative evaluations, encompassing four commonly employed ratios, indicated patellar alta, except for the Insall–Salvati index, which remained within the normal range. In instances of a “Cyrano”-type patella, where the deformity affects overall length but not the articular surface, measurements such as the Insall–Salvati ratio based on the total length of the patellar sagittal plane generate inaccurate results. Given its ability to facilitate direct comparison between preoperative and postoperative patellar heights, we deemed it more accurate and practical to measure patellar heights using the Caton–Deschamps index for cases involving a long-nosed patella.

The standard TT–TG distance is approximately 14.29 standard deviation (SD) ± 2.11 mm in men and around 12.92 ± 0.86 mm in women [18]. A higher TT–TG distance often results in a suboptimal patellar trajectory, escalating the risk of external patellar dislocation. When it reaches or exceeds 20 mm, it represents a pathological condition necessitating intervention [7]. A commonly employed surgical approach involves tibial tuberosity distalization. In this case, the TTO procedure incorporated both medialized displacement and distalization. This not only facilitates the physiological restoration of the TT–TG distance but also achieves a reduction in patellar height, contributing to the dynamic reconstruction of the MPFL [32]. The primary strategy to establish coronal balance in the knee involves either lateral patellofemoral ligament release or simultaneous combined MPFL reconstruction, aiming to achieve equilibrium in soft tissue tension on both sides of the knee. Achieving and controlling this balance can be challenging, often relying on the surgeon’s expertise. Our approach involved conducting an additional arthroscopic exploration after TTO and dynamic MPFL reconstruction to evaluate the anatomical trajectory of the patella following the combined surgical procedure (Fig. 4D). The occurrence of rotational abnormalities in the femur and tibia has been documented to reach up to 50% in the pediatric population [31]. Torsional issues tend to naturally resolve with age, resulting in only a small percentage of older children experiencing cosmetic and functional challenges. Tibial torsion and femoral torsion lead to malalignment of the patellofemoral joint forces, which causes patellar instability and eventually manifests itself in pain and even dislocation symptoms [22]. Tibial torsion exceeding 30° serves as the primary criterion for tibial de-rotation osteotomy according to most authors [2]. In instances where abnormal femoral rotation is present, prevailing evidence indicates that a singular correction of the tibia is generally satisfactory in the majority of cases [28]. Proximal tibial osteotomy is a viable option for addressing patella alta and correcting the TT–TG distance, along with simultaneous correction of tibial rotation in adults [15]; however, its application in adolescents may pose a risk to the growth of the epiphyseal plate owing to potential disturbances in skeletal development. Therefore, when dealing with patients with immature epiphyseal development, the direct correction of patellofemoral malalignment through the application of the TTO procedure is less likely to disrupt the physis. In this adolescent, the sulcus angle and trochlear depth, significant factors in patellofemoral instability, were 129.3° and 6.4 mm, respectively. The typical range for sulcus angle value is 135 ± 10°. If the sulcus angle exceeds 145°, it indicates a misalignment in the patella’s movement along the femoral trochlea, potentially leading to patellofemoral instability.

The combination of TTO with MPFL reconstruction demonstrates efficacy in addressing adolescent patellofemoral instability by adjusting both soft tissue and bone structure [6]. The safety of the combined procedure is favorable. Systematic analysis of patients undergoing combined MPFL reconstruction and TTO surgery showed a mean patient age of 16.1 years and a mean postoperative follow-up of 5.6 years [19]. The analysis revealed no evidence of deformity growth or axial excursion. Moreover, there were no significant differences in tibial and femoral lengths compared with the healthy side, except in cases where patients experienced epiphyseal diaphyseal failure, leading to decreased posterior tibial slope after the surgery. Indications for combined TTO and MPFL reconstruction surgery should not only consider the higher Caton–Deschamps index but also the longer TT–TG distance. Tibial tubercle osteotomy encompasses distalization, aimed at normalizing the Caton–Deschamps ratio to 1.0, and medialization, structured to achieve a TT–TG distance of less than 18 mm while avoiding excessive overmedialization. Bartsch’s findings [3] indicated that distalization of the tibial tuberosity may not be required for mild patella alta exhibiting patellar dislocation with a Caton–Deschamps index ranging from 1.2 to 1.4. However, the current patient presented with a high Caton–Deschamps ratio of 1.7, necessitating both distalization and medialization. The primary approach to addressing lateral patellar dislocation involves undergoing medial patellofemoral ligament reconstruction surgery. This procedure, as documented in literature, serves to not only restore the function of the medial patellofemoral ligament, establishing a stable mechanical balance between the medial and lateral soft tissue [4], but also effectively reduces the patellar height through MPFL reconstruction [11] [27]. Vampertzis *et al*. [29] noted that patella alta and recurrent patellar dislocation could result in meniscus injury or anterior cruciate ligament tear. Therefore, careful consideration should be given to assessing the status of the meniscus and cruciate ligaments in individuals with a high patellar position. In this case, preoperative MRI evaluation and intraoperative arthroscopic exploration revealed no evidence of damage to the meniscus, anterior cruciate ligament, or posterior cruciate ligament. Dynamic reconstruction offers advantages such as eliminating the need for intraoperative fluoroscopy and femoral-side fixation, particularly beneficial for younger adolescents with growth plate bones, and prevents injuries to the physis [21]. This approach also helps minimize radiation exposure for both the patient and the surgeon, prevents over-constraining of lateral patellofemoral joint [12], and eliminates the need for a femoral fixation implant.

Initially, this patient was suspected of having Osgood–Schlatter disease (OSD) and underwent physiotherapy. OSD is positively correlated with patella alta, likely due to strong quadriceps muscle pull [13]. Patients with Osgood–Schlatter disease manifest disrupted irregular patellofemoral alignment, with affected adolescents displaying increased quadriceps angles [26]. However, this abnormal quadriceps angle alone is insufficient to precipitate external patellar dislocation. Subsequent confirmation of the diagnosis revealed the initial OSD diagnosis to be inaccurate. The key distinction lies in the pain point, where OSD manifests at the tibial tuberosity, while long-nosed patella presents at the infrapatellar pole. Despite the proximity of the pain locations, a detailed clinical inquiry and physical examination are essential to avoid misdiagnosis.

### Limitations

This study presents a single clinical case without the inclusion of a control group, which limits the generalizability of the findings. In addition, it is important to note that the nonsurgical treatment attempted prior to the surgical intervention was unsuccessful. Owing to the exceptional infrequency of cases where Cyrano patella coexists with patellar instability, existing literature predominantly presents unilateral discussions on either Cyrano patella or patellar instability, with a notable absence of a comprehensive review encompassing the coexistence. The primary deliberation in the decision-making process for this case is the selection between a single-combined procedure and staged procedures, which lacks a meticulous evaluation of the merits and demerits of each choice. In addition, the determination of the surgical modality, particularly the choice between dynamic MPFL reconstruction and static reconstruction, is heavily influenced by the surgeon’s clinical experience and accumulated expertise.

Dynamic MPFL reconstruction was proposed as a reproducible and safe technique, especially in young patients, to avoid the femoral drill hole, reduce the number of necessary implants, ensure adequate patellofemoral contact pressure, and minimize fluoroscopy exposure [4, 24]. However, we acknowledge that this technique is unable to anatomically replicate the normal vector and function of MPFL. After the report of an increased complication rate from another orthopedic center [12], the technique was discontinued by the authors. Furthermore, the procedure did not involve direct intervention for tibial and femoral torsion, which remains a limitation in the comprehensive management of the condition. Confirmation of its efficacy requires long-term follow-up.

## Conclusion

The presentation of a symptomatic Cyrano patella coexists with patellofemoral instability. The surgical removal of the elongated inferior pole, combined with the realignment of the patellofemoral joint in both coronal and sagittal planes, effectively alleviated pain and improved knee joint stability. Surgical interventions that combined inferior pole osteotomy of the patella, TTO, and MPFL reconstruction ultimately contributed to the resolution of this rare condition. The final follow-up evaluations demonstrated significant improvements in KOOS, Lysholm Score, and Kujala Score. In addition, the sagittal plane deformity of the patellar shape can significantly influence the measurement of patellar height. Therefore, the Install–Salvati index does not accurately determine the patellar height in cases with an elongated “nose” of the patella.

## Data Availability

The data used and analyzed during the current study are available from the corresponding author on reasonable request.

## Abbreviations

MR: Morphology ratio
MPFL: Medial patellofemoral ligament
KOOS: Knee Injury and Osteoarthritis Outcome Score
ROM: Range of motion
CT: Computed tomography
TT-TG: Tibial tubercle trochlear groove
MRI: Magnetic resonance imaging
TTO: Tibial tuberosity osteotomy
OSD: Osgood–Schlatter disease
